# An inactivated nuclease-like domain in RecC with novel function: implications for evolution

**DOI:** 10.1186/1472-6807-5-9

**Published:** 2005-06-28

**Authors:** Daniel John Rigden

**Affiliations:** 1 School of Biological Sciences, University of Liverpool, Crown St., Liverpool L69 7ZB, UK

## Abstract

**Background:**

The PD-(D/E)xK superfamily, containing a wide variety of other exo- and endonucleases, is a notable example of general function conservation in the face of extreme sequence and structural variation. Almost all members employ a small number of shared conserved residues to bind catalytically essential metal ions and thereby effect DNA cleavage. The crystal structure of the RecBCD prokaryotic DNA repair machinery shows that RecB contains such a nuclease domain at its C-terminus. The RecC C-terminal region was reported as having a novel fold.

**Results:**

The RecC C-terminal region can be divided into an alpha/beta domain and a smaller alpha-helical bundle domain. Here we show that the alpha/beta domain is homologous to the RecB nuclease domain but lacks the features necessary for catalysis. Instead, the domain has a novel function within the nuclease superfamily – providing a hoop through which single-stranded DNA passes. Comparison with other structures of nuclease domains bound to DNA reveals strikingly different modes of ligand binding. The alpha-helical bundle domain contributes the pin which splits the DNA duplex.

**Conclusion:**

The demonstrated homology of RecB and RecC shows how evolution acted to produce the present RecBCD complex through aggregation of new domains as well as functional divergence and structural redeployment of existing domains. Distantly homologous nuclease(-like) domains bind DNA in highly diverse manners.

## Background

The largest evolutionary superfamilies of proteins cover such a large range of sequence space that the relationships shared by members may not be apparent by standard means of sequence comparison, and hence are often only recognized after structural determinations. Such has frequently been the case for the PD-(D/E)xK superfamily of nucleases. Within the superfamily, structures were first obtained for four restriction enzymes, of such diverse sequences that they were initially assumed not to share homology (reviewed in [1]). Since then structures have confirmed distant and often unexpected homologies of those four with many other restriction enzymes, as well as exo- and endo-nucleases involved in such diverse cellular processes as DNA repair [2], transposition [3], Holliday junction resolution [4] and recombination [5].

The unifying catalytic site characteristic of the superfamily is the presence of one or more catalytically essential divalent cations [6,7]. The conserved acidic residues of the PD-(D/E)xK motif, which can be separated by any number of residues, bind one metal cation while the conserved lysine residue is involved in positioning water suitably to attack the DNA backbone. In some lineages of the superfamily variation on this classical motif is apparent in the substitution of the second acidic residue by a catalytically essential His residue (2), or in the migration of the second acidic residue [8] or the lysine residue [9] to other parts of the fold. Irrespective of this variation, the catalytic site is placed at one edge of the core four or five-stranded β-sheet at the heart of the α /β domain structure [1,6,7]. While an overwhelming majority of the superfamily contain one of these catalytic site variants some interesting exceptions have been noticed. Thus, while clearly containing a PD-(D/E)xK superfamily-like domain structure [10], the tRNA splicing endoribonuclease EndA, has evolved an unrelated catalytic site on the opposite side of the fold to the conventional site [11]. A catalytically inactive version of the fold has also been seen in the N-terminal domain of *S. cerevisiae *RPB5, an RNA polymerase subunit, where evidence suggests that it functions in protein-protein interactions [12].

Although extremely diverse in structure and sequence, modern sequence comparison methods have played their part in elucidating the full range of PD-(D/E)xK superfamily members [9,13-15]. Nevertheless, structure determinations and structure-informed bioinformatics [16] will continue to be crucial in this most diverse of superfamilies. Some five years ago it was predicted that the nuclease activity associated with the C-terminus of RecB [17] resulted from the presence of a domain homologous to that of λ-exonuclease, despite RecB not possessing a PD-(D/E)xK motif [13,14]. This prediction has been recently confirmed with the crystal structure determination of the structure of the RecBCD heterotrimer [18]. This remarkable complex (see [18] and references therein) which functions to process double-stranded breaks in DNA, contains two distinct helicase activities, contributed by RecB and RecD. Also present is a catalytically inactive subunit, RecC. Among its proposed roles is recognition of the Chi DNA sequence [18]. Remarkably, twin helicase(-like) motor domains (canonically named 1A and 2A) are present in all three subunits, although those in RecC are inactivated and only those in RecB and RecC contain α-helical insert domains in each motor domain (named 1B and 2B, respectively). As mentioned, the helicase domains of RecB are followed by a PD-(D/E)xK superfamily nuclease domain 3. In contrast, the C-terminal 'domain 3' of RecC was reported as being of novel fold [18].

Here we show that the C-terminal region ('domain 3') of RecC can actually be dissected into two domains, the first of which is clearly related to PD-(D/E)xK superfamily nuclease domains (hereafter called simply nuclease domains) and particularly to the corresponding domain of RecB. The nuclease-like domain of RecC is inactivated and therefore possesses not even the metal-ligating residues of the PD-(D/E)xK motif. Instead, it carries out a function not hitherto observed in the superfamily, providing an aperture through which one strand of newly split DNA duplex is fed. Comparisons show that nuclease(-like) domains are extraordinarily versatile in their mode of interaction with duplex DNA. Characteristics of the RecC nuclease-like domain show that RecB and RecC share a common ancestor and reveal how evolution has progressed by sequential addition of domains to the C-terminus, as well as by altering function of, and repositioning of, existing domains.

## Results and discussion

### An unsuspected nuclease-like domain in RecC

**Figure 1:**
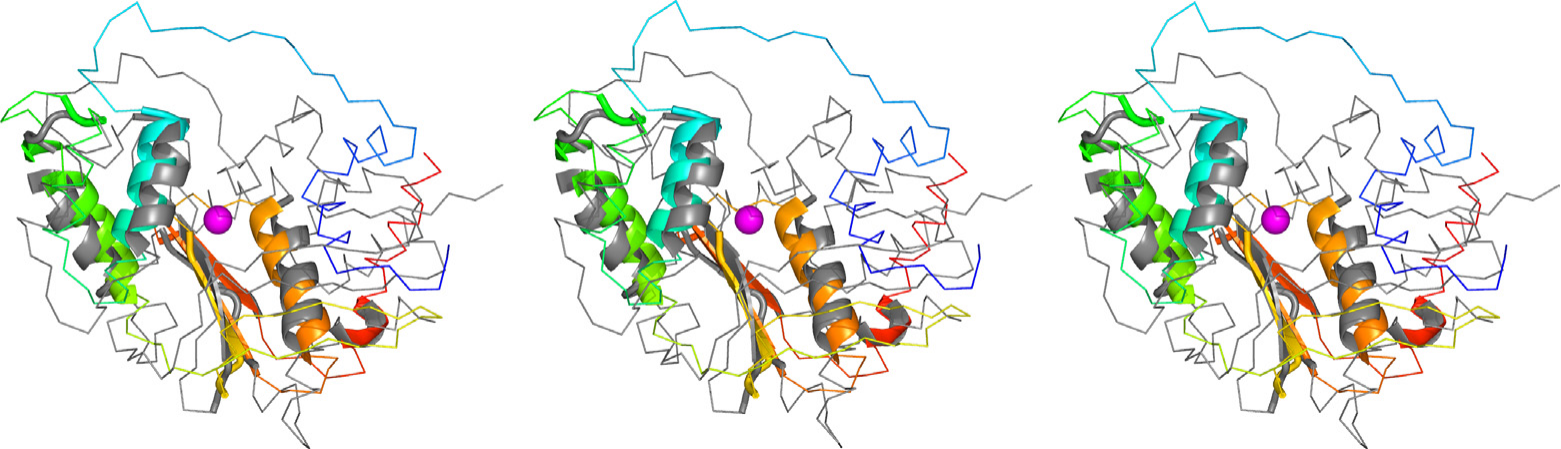
**Stereo structural superposition of the nuclease(-like) domains of RecB and RecC produced with LSQMAN [29]. **RecB is coloured grey while RecC, is coloured in a spectrum from blue (N-terminus) to red (C-terminus). Structurally superposed regions are shown in cartoon representation (rms deviation of 2.19Å for 71 Cα atoms), other parts as a Cα trace. The calcium ion bound to RecB is shown as a magenta sphere.

**Figure 2:**
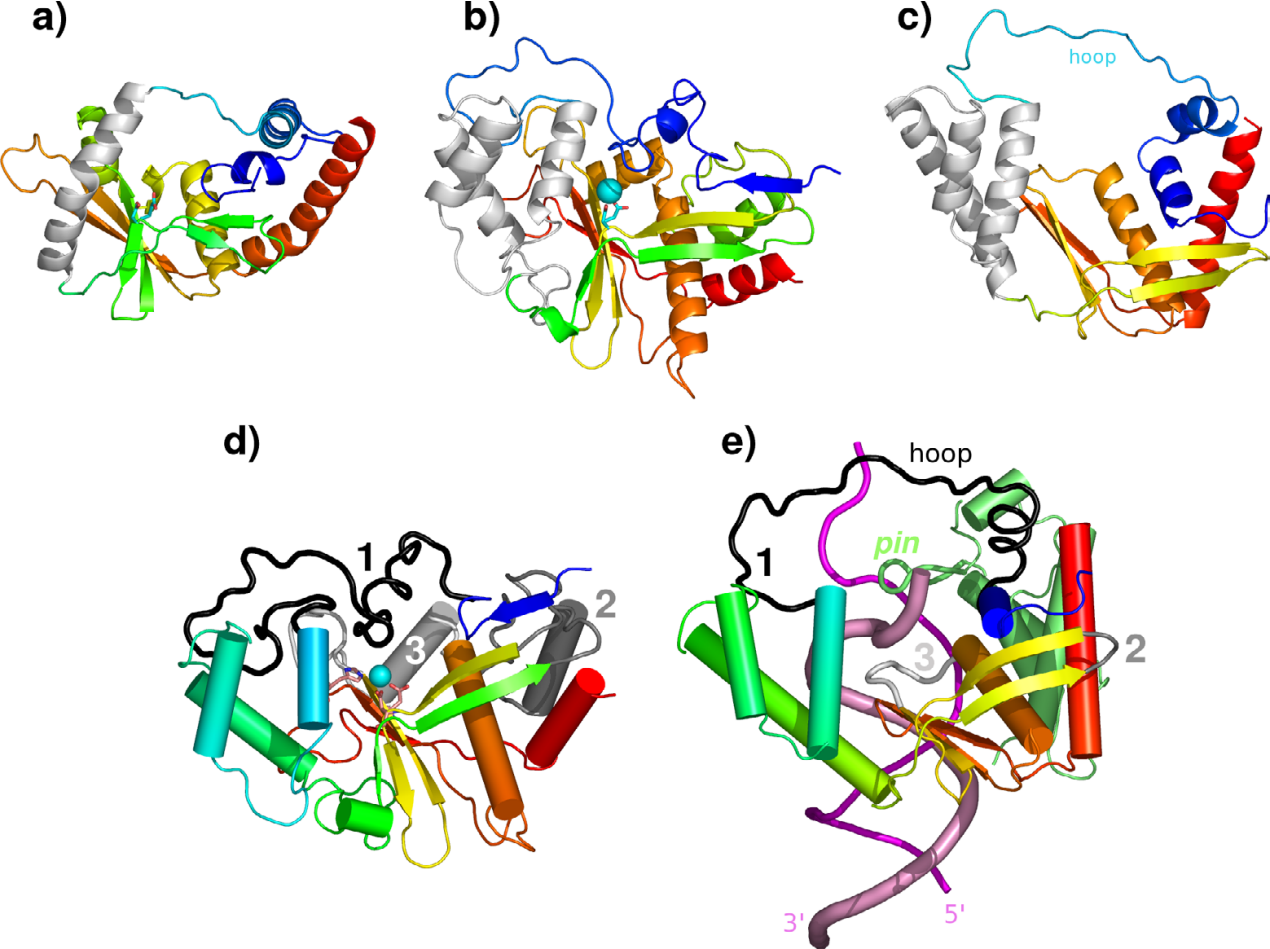
**Comparisons of structurally aligned nuclease(-like) domains in λ-exonuclease, RecB and RecC. **The comparison in a)-c) shows how a single helix in λ-exonuclease (PDB code 1avq; [5]) (a) has been replaced by superimposable α-helical bundles in RecB (b) and RecC (c) (PDB code 1w36; [18]), indicating a more recent shared ancestor of the latter pair. The regions in question are shown as light grey. The remainders of the molecules are coloured in a spectrum from blue (N-terminus) to red (C-terminus). In a) and b), two acidic, metal-ligating residues drawn as sticks mark respective catalytic sites. RecB and RecC are compared in more detail in d) and e), respectively, again coloured from blue to red with the exception of labelled key regions 1 (black), 2 (dark grey) and 3 (grey). Bound metal is shown in b) and d) as spheres while e) additionally shows DNA (shades of pink) and the domain 4 of RecC, coloured uniformly lime green with its pin structure labelled. The DNA strand that penetrates the hoop provided by RecC is shown as a broader cartoon. The RecC "hoop" region (see text for details) is labelled in c) and e) and DNA strand termini are labelled in e).

Domain 3 of RecC has been described as being of novel fold [18]. Structural examination suggested that it could, in fact, be divided into two domains, an α/β domain and a C-terminal all α-helical domain. Although the division was made by eye initially, analysis with Protein Domain Parser [19] produced a result that differed by just two residues. When the α/β domain (comprising residues 828–1033) was submitted to DALI [20], the most closely related structure in the database was reported as phosphoserine phosphatase but in second place was λ-exonuclease (PDB code 1avq; [5]). A root mean squared (rms) deviation between the third RecC domain and λ-exonuclease of 4.2Å for 121 Cα atoms was obtained (yielding a DALI Z score of 4.1). λ-exonuclease is the nearest structural neighbour to the nuclease domain of RecB [18]. For that pair, 131 Cα atoms can be superimposed with an rms deviation of 3.5 Å (Z score of 6.2). From these data and visual inspection (later additionally supported by PSI-BLAST results – see below), it is clear that the third RecC domain is a relative of the nuclease domain common to RecB and λ-exonuclease (Figures 1 and 2). Notably, the further division of the C-terminal RecC 'domain 3' into two domains was essential for this relationship to become apparent. In contrast, the fourth, α-helical bundle domain of RecC has no close neighbours in the present database.

**Figure 3:**
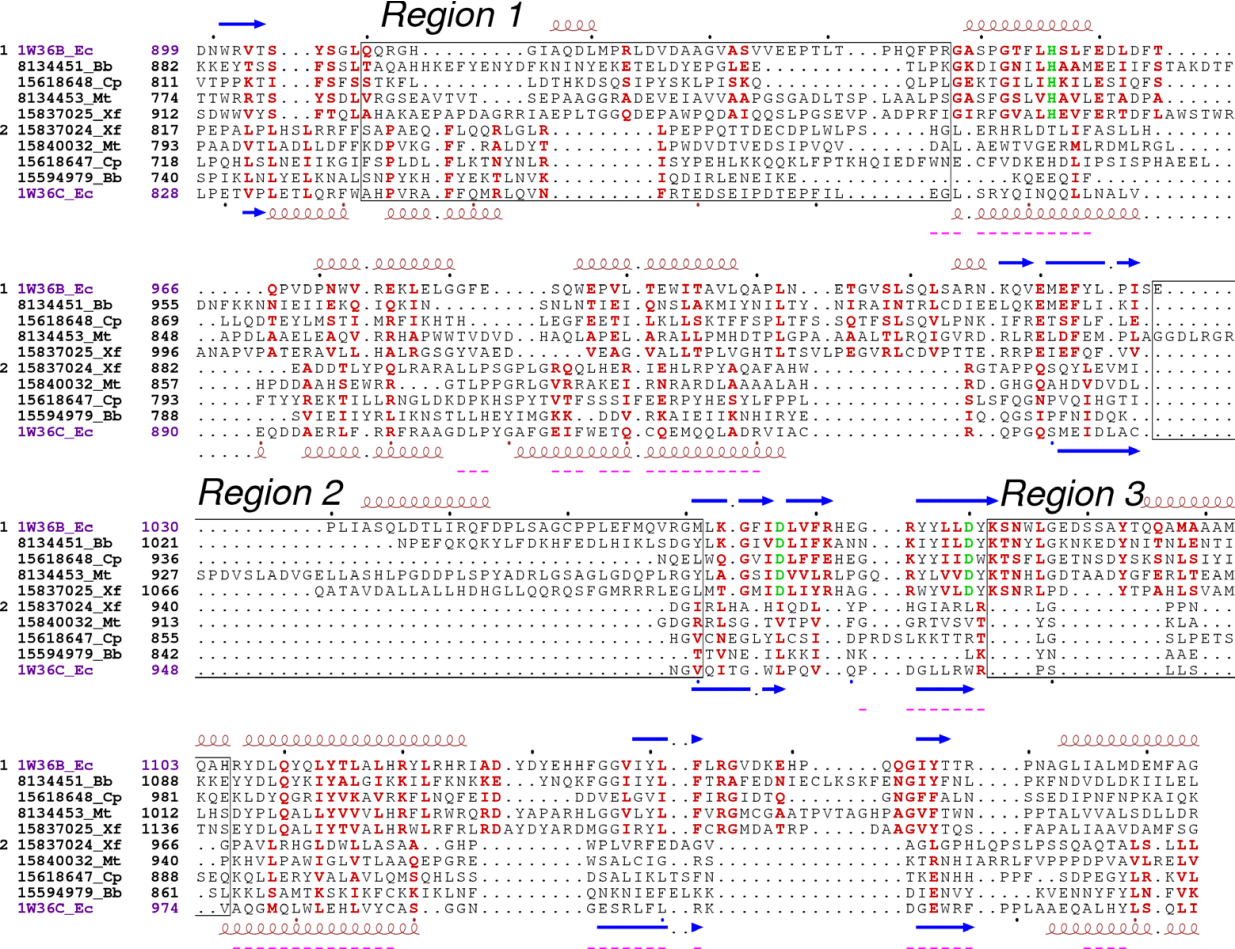
**Structure-based sequence alignment of the nuclease(-like) domains of RecB and RecC. **Nuclease(-like) domain sequences of RecB (above, group 1) and RecC (below, group 2) were chosen from diverse representative species and extracted from complete alignments of COG database [31] entries for RecB or RecC. Purple indicates the *E. coli *sequences crystallized as the RecBCD complex (PDB code 1w36; [18]). Other sequences are labelled with Genbank numbers and sequence codes Bb, *Borrelia burgdorferi*; Cp, *Chlamydophila pneumoniae*; Mt, *Mycobacterium tuberculosis*; Xf, *Xylella fastidiosa*. Red colouring indicates conservation within each group while green is used for three important catalytic residues of RecB – H956, D1067 and D1080 [18]. Elements of regular secondary structure are shown above (RecB) and below (RecC) the alignment, where spirals represent α-helices and arrows β-strands. The three key regions (numbered 1–3) involved in adaptation of the RecC nuclease-like domain to its new function, as discussed in the text (see also Fig 2), are boxed and labelled. Purple underlining indicates zones that can be simultaneously structurally aligned (rms deviation of 2.19Å for 71 Cα atoms).

The nuclease fold common to λ-exonuclease, RecB and now RecC is found in a wide variety of exo- and endonucleases, from restriction enzymes to Holliday junction resolvases, and enzymes of DNA repair [14]. Within the superfamily, conserved motifs vary with family, but all centre on acidic residues involved in binding the divalent metal cation typically required for catalysis [6,7]. These residues are the sole residues conserved across almost the whole superfamily. A calcium ion bound to RecB in the crystal structure [18] marks the binding site for the essential metal while in λ-exonuclease, soaking in manganese has revealed the corresponding site [5]. A metal-binding site, like those shown in Figure 1, is not present in RecC (Figure 3). Indeed, the overall sequence identity between the RecB and RecC sequence segments shown in Figure 3 is just 2–11 %. Thus, just as domains 1 and 2 of RecC are inactivated helicase domains [18], so its domain 3 is an inactivated nuclease.

Interestingly, comparison of the nuclease domains of RecB, RecC and λ-exonuclease shows that the Rec subunits clearly share a more recent ancestor than the common ancestor of all three structures. As Figures 1 and 2 show, a single helix present in λ-exonuclease is replaced in both RecB and RecC by a three-helix α-helical bundle. This bundle is not present in the more distant relatives of λ-exonuclease highlighted by the CE server [21] such as archaeal Holliday junction resolvase, tRNA endonuclease and the PvuII restriction enzyme. Curiously, the degree of structural superposition that can be achieved between the RecB and RecC nuclease domains and λ-exonuclease suggests no closer relationship between the former pair. For example, 71 Cα atoms of RecB nuclease domain may be superimposed on their equivalents in RecC to produce an rms deviation of 2.19 Å. In comparison, 82 Cα atoms of RecB superimpose on equivalents of λ-exonuclease with a lower rms deviation of 1.71Å. However, the superimposable three-helix α-helical bundle shared only by RecB and RecC (Figures 1 and 2) show that they are more closely evolutionarily related to each other than to other homologous structures. The closer structural superposition of RecB and λ-exonuclease seems likely to arise from their shared nuclease activity, while RecC has evolved a different function.

### Novel function of the nuclease-like domain in RecC

As mentioned, nuclease domains as represented in the present PDB are extremely diverse in sequence but share conserved residues that bind essential metal ions and are almost invariably catalytically active. The recognition of the third domain of RecC as an inactivated nuclease domain highlights a wholly unexpected new function for a non-catalytic but clearly nuclease-like domain. As shown in Fig 2e, the nuclease-like domain of RecC provides a hoop through which a single strand of the newly separated DNA duplex is passed. The hoop is the entrance to the 5' channel leading to RecD in the RecBCD complex [18]. The pin responsible for separating the two DNA strands consists of a loop extending out of the α-helical bundle domain 4 of RecC.

Structural comparisons show that a series of three structural adaptations have been required in RecC in order to achieve this novel ssDNA-hoop function. These involve three regions of sequence marked on Figures 2 and 3. Region 1 comprises a long linker sequence between the extended structure that starts the domain and the three helix α-helical bundle subdomain. This linker region is very poor in regular secondary structure and adopts dramatically different conformations in the two domains. Significant sequence identity between RecB and RecC seems absent in the region. In RecB this linker lies along the surface of the remainder of the domain. In dramatic contrast, region 1 in RecC has few contacts with the rest of the domain (although it contacts other parts of RecC – see below) and forms most of the rim of the hoop through which ssDNA is passed. Region 2 is the connection between the two strands forming an antiparallel β-sheet. In *E. coli *RecC the connection is a minimal β-turn and connections in other RecC sequences are also very short (Figure 3). In contrast, Region 2 in RecB is usually much larger, tracing out, in the *E. coli *RecB structure, an 11-residue α-helix and a substantial stretch lacking regular secondary structure. Structure comparison shows the reason for the short connectors in RecC (Figure 2); larger connectors occupy the same space as the fourth domain of RecC. Thus, a larger connector would be incompatible with a RecC-style pin domain. Region 3, providing the connector between a β-strand and an α-helix, is again larger in RecB than in RecC and again contains an α-helix in RecB. Here the reason for the shorter connector in RecC is even more fundamental; were it to have the longer connector of RecB, the aperture whereby ssDNA passes through the RecC nuclease-like domain would be sterically obstructed.

### DNA interactions with nuclease and nuclease-like domains

Unfortunately, no structure of λ-exonuclease in complex with DNA is yet available. However, other enzymes sharing the same fold, including many type II restriction enzymes, have been crystallized in complex with DNA. Therefore, DNA-bound structures were sought for the enzymes identified as closest structural neighbours for λ-exonuclease by the CE server [21]. This analysis pinpointed the restriction enzyme PvuII (PDB code 1pvi; [22]) and the vsr exonuclease (PDB code 1odg; [23]) involved in repair of bacterial G:T mismatches. Further analysis (not shown) showed that the mode of binding of DNA to Pvu II was, in fact, typical of many restriction enzymes, irrespective of dimeric *vs *tetrameric quaternary state and of differing modes of dimerization.

**Figure 4:**
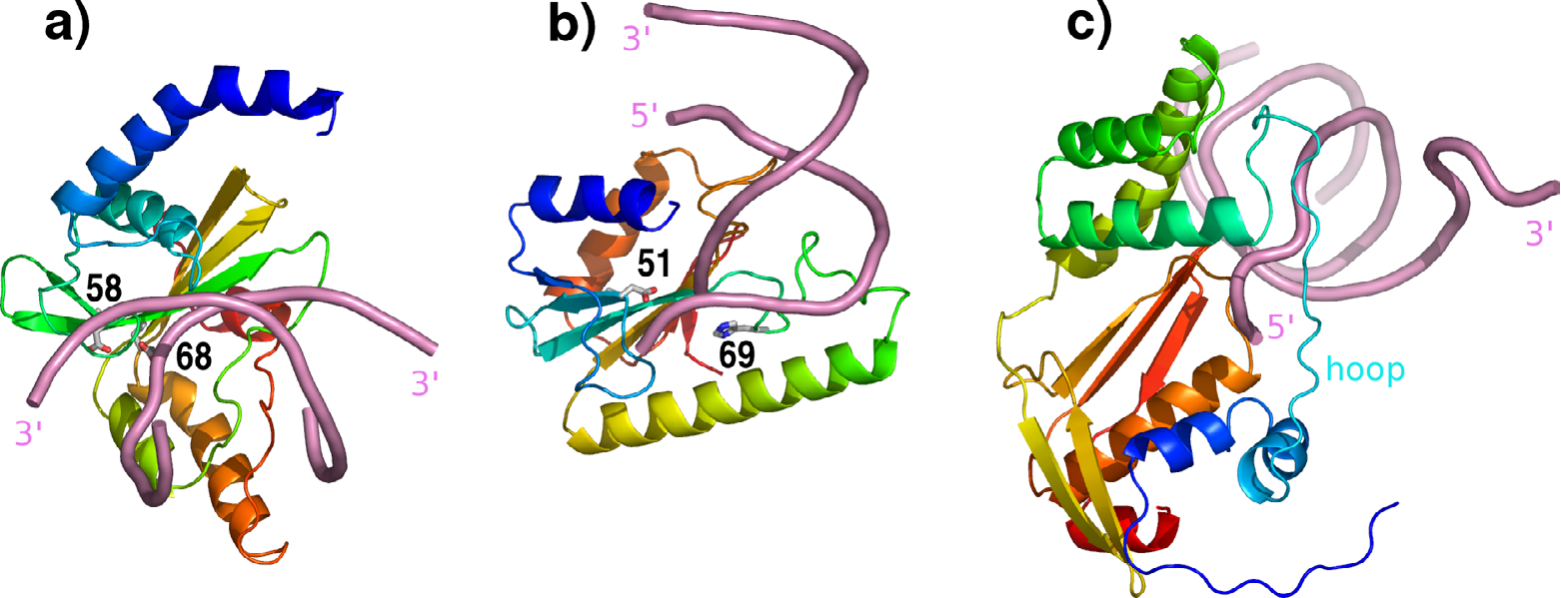
**Comparison of modes of DNA binding to superimposed nuclease(-like) domains. **The domain structures are those of a) PvuII (PDB code 1pvi; [22] b) vsr exonuclease (PDB code 1odg; [23]) and c) RecC (PDB code 1w36; [18]). Protein chains are coloured in a spectrum from blue (N-terminus) to red (C-terminus) while DNA is coloured uniformly pink. In order to illustrate the approximate locations of the catalytic sites, selected catalytic residues are shown for PvuII (D58 and E58) and vsr exonuclease (D51 and H69). DNA termini are labelled, as is the "hoop" in RecC.

Remarkably, as shown in Figure 4, the axes of duplex DNA binding to PvuII and to vsr exonuclease are almost orthogonal, a difference that seems to have escaped notice. The catalytic sites of both enzymes, although differing in detail, are similarly placed at one edge of the β-sheet, defining the 'front' of catalytic nucleases. Most unexpectedly, the inactivated nuclease-like domain of RecC which also, in the context of RecBCD, binds duplex DNA, prior to strand splitting by the fourth domain, does so in a completely novel manner. First, the axis of the bound duplex DNA is approximately orthogonal to both PvuII and vsr exonuclease modes. Secondly, the binding involves the 'back' of the domain; only a single strand of the DNA arrives at the 'front' side after passing through the aperture (Figure 4). These results make clear that few assumptions can be made regarding modes of DNA binding by nuclease(-like) domains in the absence of experimental data such as structures in complex with DNA.

### Homology of RecB and RecC

The observation of inactivated helicase-like domains in RecC was not considered reason enough to propose the existence of homology between RecB and RecC extending over their whole length [18]. Indeed, both sequence and structural comparisons at first suggest that RecB more closely resembles other helicases than it does RecC. For example, in the results of PSI-BLAST [24] starting with *E. coli *RecB, PcrA, another helicase that contains large helical-insert domains in each helicase domain [25], appears as a significant hit (e = 6 × 10^-9^) in the results of the first iteration. In contrast, using an e-value cut-off of 0.0001 four iterations are required before RecC sequences, including that of *E. coli *RecC, appear among the significant hits. While the BLAST alignments centred on the helicase(-like) domains the C-terminal nuclease(-like) domains were sometimes matched, although PSI-BLAST runs of the nuclease domain of RecB failed to hit the nuclease-like domain of RecC, and *vice versa*. Similarly, structural comparisons show that both helicase domains and both α-helical insert domains of RecB are more similar to their counterparts in PcrA than to the corresponding RecC domains (not shown). Nevertheless, the clear homology of the RecB and RecC nuclease(-like) domains, evident in their common three α-helical bundle (see above) strongly suggests that RecB and RecC share a more recent common ancestor than they have in common with other extant helicases. How then to explain the apparently closer relationship of RecB with PcrA than with RecC? As was proposed for the nuclease(-like) domains (see above) it seems like the dramatic functional differences between corresponding RecB and RecC domains are responsible. As discussed above, the RecC nuclease-like domain is significantly shorter than the RecB nuclease domain in two key regions, each associated with its new role as provider of an ssDNA hoop. Thus, it seems plausible that the maintenance of helicase activity by the helicase domains of PcrA and RecB is responsible for their apparently closer relationship, the structural changes accompanying evolution of the helicase-like domains in RecC for new roles having obscured their more recent shared ancestry with RecB.

**Figure 5:**
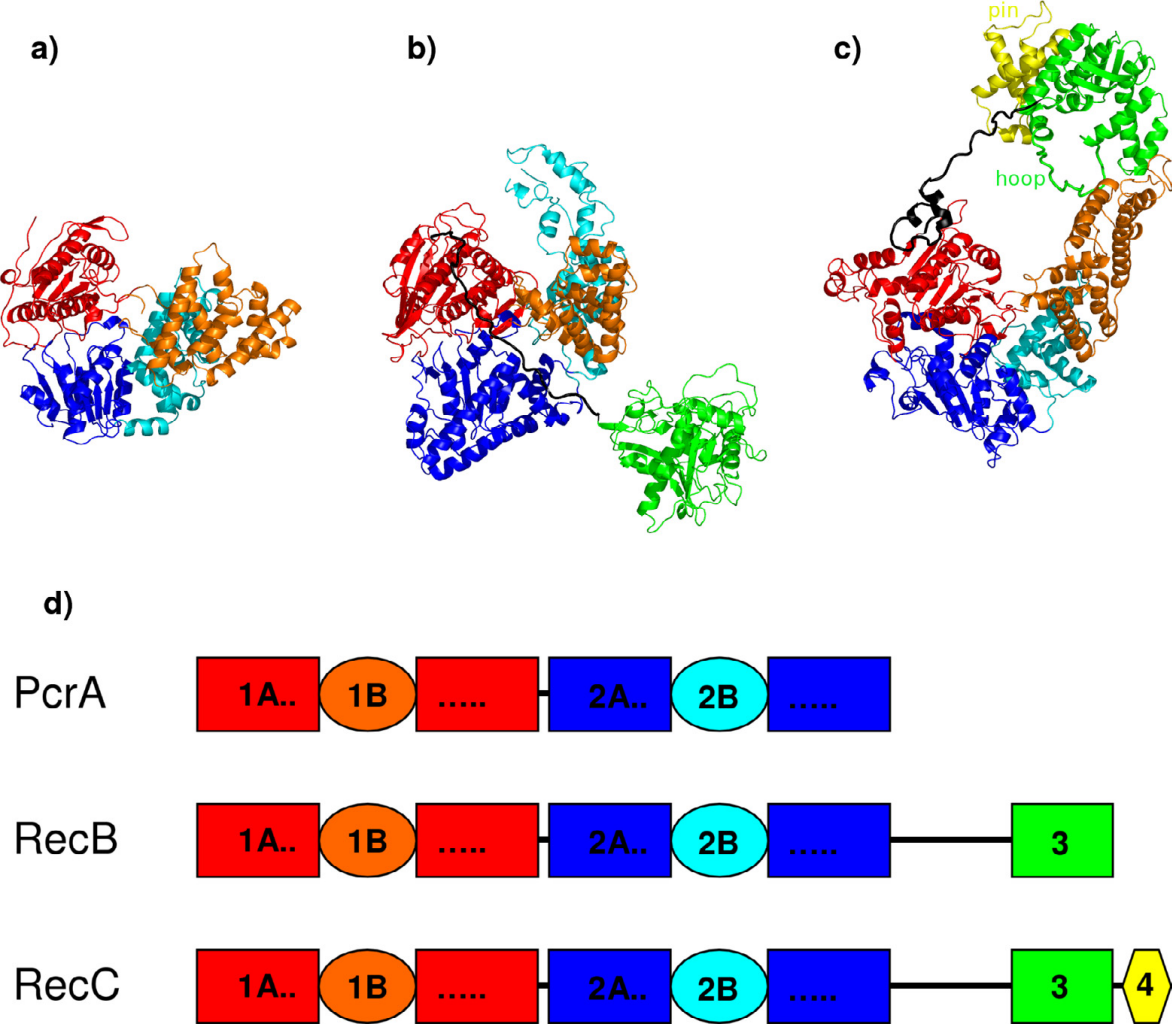
**Domain comparison of PcrA, RecB and RecC. **The structures of a) substrate-complexed PcrA (PDB code 3pjr; [38]), b) RecB and c) RecC (PDB code 1w36; [18]), are superimposed using domain 2A. The domains 1A are coloured red, while orange is used for 1B, blue for 2A, cyan for 2B, green for 3 and yellow for 4. The same colours are used in the schematic diagram d) which illustrates how evolution progressed through addition of domains. The "pin" and "hoop" in RecC (see text for details) are labelled. In PcrA the residues defining the starts of the various (sub-)domains are 1B, 92; 1A continuation, 218; 2A, 287; 2B, 385; 2A continuation, 553. In RecB the corresponding residue numbers are 1B, 151; 1A continuation, 349; 2A, 446; 2B, 583; 2A continuation, 729; linker, 870; 3, 900. In RecC they are 1B, 79; 1A continuation, 208; 2A, 329; 2B, 443; 2A continuation, 649; linker, 784; 3, 828; 4, 1034.

The recognition of homology between RecB and RecC, and the dissection of their domains leads to an interesting comparison with PcrA. In PcrA, a duplication of an ancestral RecA-like domain, already containing an all α-helical insert domain, is evident [25]. In RecB a long linker region and following nuclease domain have been added to the PcrA template (Figure 5). A further domain addition has occurred in RecC, that of the small C-terminal α-helical bundle domain that contributes the duplex-splitting pin. This picture of aggregation of novel functionality through domain addition is complemented by alterations in function of homologous domains. Thus, as described, the nuclease-like domain of RecC continues to bind duplex DNA, but using a different surface of the domain, as well as providing the entrance to the 5' ssDNA channel leading to RecD. This modification is paralleled in the helicase-like domains by a change from catalytic helicase activity to Chi sequence recognition ([18] and references therein). The α-helical inserts into the helicase(-like) domains also have different functions in RecB and RecC [18], including, in the case of the RecC domain 1B binding to the RecC nuclease-like domain and the rim of its ssDNA aperture (Figure 5). Although homologous, the structural comparison of complete RecB and RecC subunits shows large differences in relative domain orientations and positions, most dramatically with regard to the position of the nuclease(-like) domains relative to the helicase(-like) domain cores (Figure 5).

There is an interesting parallel to be drawn between RecBCD and AddAB (also known as RexAB), a different DNA repair system found in Gram positive bacteria where RecBCD is lacking (see [26] for a review). AddA and AddB also appear homologous and each possesses helicase and nuclease motifs. Within AddAB, it is AddB that recognises the Chi sequence and therefore is the counterpart of RecC in RecBCD. Most interestingly, however, both the nuclease domains of AddA and AddB appear to be active [27]. The AddAB system may therefore resemble an evolutionarily intermediate stage, through which the RecBCD machine passed before inactivation of the RecC nuclease domain and recruitment of RecD.

In summary, the improved domain dissection of RecC presented here and its ramifications enhance our understanding of the evolutionary processes responsible for the remarkable DNA processing machinery that is the RecBCD complex [18]. It is now even more apparent that relatively straightforward addition of modular functionality has been accompanied by quite dramatic functional evolution of homologous domains.

## Methods

Protein structures were retrieved from the Protein Databank (PDB; [28]). Protein structural superpositions were obtained at the CE [21] and DALI [20] servers and by using the program LSQMAN [29]. Structural relationships were also explored in the SCOP database [30]. Protein structure visualization employed O [31] and PyMOL [32], the latter also being used for production of figures. Iterative database searches were carried out using PSI-BLAST [24]. Sequences were retrieved from the COG [32] entries for RecB (COG1074) and RecC (COG1330). Maximally diverse representatives were chosen using JALVIEW [34] which was also used for general sequence manipulation. Protein sequence alignment was carried out using MUSCLE [35] and T-COFFEE [36]. Formatting of sequence alignments was done with ESPRIPT [37] using default options for colouring of sequence conservation.
